# Dexmedetomidine protects against renal ischemia and reperfusion injury by inhibiting the JAK/STAT signaling activation

**DOI:** 10.1186/1479-5876-11-141

**Published:** 2013-06-09

**Authors:** Yanna Si, Hongguang Bao, Liu Han, Hongwei Shi, Yuan Zhang, Li Xu, Chenhui Liu, Jinsong Wang, Xiaobing Yang, Akbar Vohra, Daqing Ma

**Affiliations:** 1 Department of Anesthesiology, Nanjing First Hospital, Nanjing Medical University, Nanjing 210006, People’s Republic of China; 2 Department of Pathology, Nanjing First Hospital, Nanjing Medical University, Nanjing 210006, People’s Republic of China; 3 Manchester Academic Health Sciences Centre, Manchester Royal Infirmary, Manchester M13 9WL, UK; 4 Anaesthetics, Pain Medicine and Intensive Care, Department of Surgery and Cancer, Faculty of Medicine, Imperial College London, Chelsea Westminster Campus, 369 Fulham Rd, London Sw10 9NH, UK

**Keywords:** Dexmedetomidine, Ischemia and Reperfusion Injury, AG490, JAK/STAT, Renoprotection

## Abstract

**Background:**

The α_2_-adrenoreceptor agonist dexmedetomidine is known to provide renoprotection against ischemia and reperfusion (I/R) injury. However the underlying molecular mechanisms remain unclear. The purpose of this study was to investigate whether the Janus kinase and signal transducer and activator of transcription (JAK/STAT) signaling pathway plays a role in dexmedetomidine’s renoprotection.

**Methods:**

I/R model was induced by bilateral renal pedicle clamping for 45 min followed by 48 h of reperfusion in male Wistar rat. Sham laparotomy served as controls. Animals received dexmedetomidine (50 μg/kg, i.p.) in the absence or presence of atipamezole (250 μg/kg, i.p.), or vehicle (DMSO) in the absence or presence of selective JAK2 inhibitor tyrphostin AG490 (10 mg/kg, i.p.) before ischemia. Renal function, histology, apoptosis, expression of cleaved caspase 3 protein, intercellular adhesion molecule-1 (ICAM-1), monocyte chemoattractant protein-1 (MCP-1) and phosphorylations of JAK2, STAT1 and STAT3 were assessed.

**Results:**

The animals treated with either dexmedetomidine or AG490 exhibited an improved renal functional recovery, attenuated histological lesions and reduced number of apoptotic tubular epithelial cells. Either dexmedetomidine or AG490 inhibited the phosphorylations of JAK2 and its downstream molecule STAT1 and STAT3, accompanied by down-regulation the expression of cleaved caspase 3, ICAM-1 and MCP-1 proteins, and significantly ameliorated renal I/R injury.

**Conclusions:**

Dexmedetomidine protects kidney against I/R injury, at least in part, through its inhibitory effects on injury-induced activation of JAK/STAT signaling pathway. If our data can be extrapolated to clinical setting, then dexmedetomidine may therefore serve as a clinical strategy to treat/prevent perioperative renal I/R injury.

## Background

Perioperative acute kidney injury (AKI) induced by renal ischemia and reperfusion (I/R) is a common clinical event caused by reduced blood supply to the kidneys being compromised during major cardiovascular surgery [1]. Despite advances in preventive strategies and supportive measures, AKI is still associated with prolonged hospitalization as well as high morbidity and mortality rates which have not decreased significantly over the past 50 years [2,3]. Vasoconstriction, oxygen-derived free radicals, loss of proximal tubular cell polarity and infiltration of adhesion molecules, which lead to impairment of cell-cell and cell-matrix adhesion structures, have been shown to be implicated in the pathogenesis of renal I/R injury (IRI) [4]. Acute inflammatory responses initiated during ischemia and reperfusion, characterized by the induction of an inflammatory cytokine cascade, expression of adhesion molecules and cellular infiltration, lead to necrosis and apoptosis of renal cells [5].

Dexmedetomidine (DEX) is among a number of prophylactic and therapeutic measures that have been used to reduce perioperative AKI [6,7]. It is a highly selective α_2_-adrenoreceptor agonist with sedative, analgesic, sympatholytic and hemodynamic stabilizing properties [8]. Recent studies suggest that dexmedetomidine has organoprotective effects, reducing cerebral, cardiac, intestinal and renal injury which can be abolished by atipamezole (Atip), an α_2_-adrenoreceptor antagonist [9-14]. The α_2_-adrenoreceptors are widely distributed in the renal proximal and distal tubules, peritubular vasculature as well as in systemic tissues [15]. Dexmedetomidine treatment has been found to inhibit vasopressin secretion, enhance renal blood flow and glomerular filtration, and increase urine output [16-18]. Dexmedetomidine also has a cytoprotective effect against renal I/R injury. The combination of these aforementioned properties may contribute to improving renal function under ischemic conditions [14,17]. However, the underlying molecular mechanisms of dexmedetomidine’s renoprotection remain unknown.

It is possible that activation of Janus kinase/signal transducer and activator of transcription (JAK/STAT) pathway is involved in the development of renal I/R injury, during which many pro-inflammatory cytokines are up-regulated [19]. The JAK/STAT pathway is composed of a family of receptor-associated cytosolic tyrosine kinases (JAKs) that phosphorylate a tyrosine residue on bound transcription factors (STATs). JAK-mediated tyrosine phosphorylation of STAT family members enables translocation of these transcription factors to the nucleus and lead to an augmentation of gene transcription [20,21]. The putative JAK2 inhibitor AG490, which induces inactivation of downstream STATs, protects against ischemia-induced acute renal injury [19]. STAT3 knockout animals have revealed the pleiotropic role of STAT3 in many organs and cell types including the heart, skin, T lymphocytes, monocytes/neutrophils, mammary epithelium, liver and neurons following ischemia [22]. It has been proven recently that STATs, present in the mitochondria, modulate mitochondrial respiration, regulate mitochondria-mediated apoptosis and inhibit the opening of mitochondrial permeability transition pores (MPTP) [23,24]. Of all the JAK/STAT pathways, JAK2 signaling through STAT1 and STAT3 are the best studied in diseases affecting the kidney. An *in vitro* study has shown that dexmedetomidine may exert a significant neuroprotective effect by involving the activation of extracellular regulated protein kinases (ERK) [25]. Interference with ERK and STAT signaling pathways may also play a role in myocardial I/R injury [20].

To the best of our knowledge, the internal mechanism linking the JAK/STAT signaling pathway and the cytoprotective effect of dexmedetomidine on renal issue following ischemia has not been identified. The aim of the current *in vivo* study was to identify the main JAK/STAT signaling pathway involved in the dexmedetomidine-induced renoprotection against I/R injury in rats.

## Subjects and methods

### Animals

Male Wistar rats weighing 250–320 g were obtained from Animal Experiment Centre, Nanjing Medical University, Nanjing, China. Animals were housed in temperature and humidity-controlled cages and allowed free access to standard rodent chow and sterile acidified water in a specific pathogen-free facility at Nanjing Medical University. This study had prior approval from the Institute Animal Ethics Committee of Nanjing Medical University and all procedures described here were performed strictly under our institutional guideline.

### Treatment protocol

A total of forty-eight animals were prepared surgically for renal I/R as previously described [26]. Rats were anesthetized using with pentobarbital sodium (65 mg/kg, intraperitoneally) and a rectal probe was inserted to monitor body temperature, which was maintained at 38 ± 1°C by a heating blanket. A midline laparotomy was performed and the abdominal cavity was fully exposed. Bilateral renal pedicles were carefully isolated without damaging the ureter and clamped by non-traumatic microvascular clamps to effect complete cessation of renal arterial blood flow. After 45 minutes, the clamps were removed to allow return of blood flow to the kidneys. Successful ischemia or reperfusion was judged by observing the change in tissue color from red to dark blue or from dark blue to bright red respectively. Renal blood flow was measured by Doppler to detect sufficient ischemia had been obtained. Middle abdominal incisions were closed in two layers and covered with antibiotic ointment when the operation finished. The animals were allowed to recover from anesthesia, remaining 48 hours in a controlled-environment room with food and water freely available. Rats in the sham group underwent laparotomy without performing renal ischemia as controls.

Animals (n = 8/group) received dexmedetomidine (50 μg/kg, i.p.) in the absence or presence of atipamezole (250 μg/kg, i.p., 30 min prior to dexmedetomidine treatment), or vehicle (containing 45% DMSO and 55% normal saline) in the absence or presence of selective JAK2 inhibitor tyrphostin AG490 (10 mg/kg, i.p., Sigma-Aldrich, St Louis, MO, USA) 30 min before ischemia. All animals were euthanised by an overdose of pentobarbital sodium at the end of the experiment.

Blood samples were obtained from the abdominal aorta at the time point of 48 h after renal ischemia and allowed to clot and centrifuged at 6000 rpm for 15 min. Serum was separated and stored at −20°C for further biomedical determination. 48 h after renal ischemia, the right kidney was snap frozen at −80°C and the left one was immediately post-fixed with 10% neutral buffered-formalin solution for 2 h at 20-25°C, dehydrated with ethanol and embedded in paraffin for further analysis.

### Histological examination

Formalin-fixed renal tissue was dehydrated, embedded in paraffin and sliced into 4 μm thick sections which were stained with hematoxylin and eosin. Histological lesion (acute tubular necrosis) was graded on a scale of 0 to 4 as follows and described by Jablonski et al. [27]: 0 = normal kidney; 1 = minimal damage (<5% involvement of the cortex or outer medulla); 2 = mild damage (5–25% involvement of the cortex or outer medulla); 3 = moderate damage (25–75% involvement of the cortex or outer medulla); 4 = severe damage (>75% involvement of the cortex or outer medulla).

### Apoptosis assay

For detection of apoptotic tubular epithelial cells, TUNEL (terminal deoxynucleotidyl transferase (TDT)-mediated digoxigenin-labelled UTP nick end labelling) assay was performed by ApoTag peroxidase *in situ* cell death detection kit (Roche, Basal, Switzerland). Briefly, 4 μm thick paraffin sections were deparaffinized, then treated with proteinase K and subsequently incubated with a mixture of nucleotides and TdT enzyme at room temperature for 1 h in a humidified atmosphere. The sections were further incubated with anti-digoxygenin conjugated to horseradish peroxidase at room temperature for 30 min. The nuclei fragments were stained using 3,3-diaminobenzidine (DAB) as a substrate for the peroxidase. As a negative control, sections were incubated by omitting the TdT enzyme. Apoptosis was also evaluated using previously defined morphological criteria [28]. These criteria include eosinophilic cytoplasm, cytoplasmic shrinkage, nuclear fragmentation, nuclear chromatin condensation, membrane-bound cellular blebbing and formation of apoptotic bodies.

### Biochemical determinations

Serum creatinine and plasma urea concentrations were measured by standard picric acid--based colorimetric kinetic assay using an automatic biochemical analyzer (Olympus AU2700, Mishima, Japan). Plasma levels of intercellular adhesion molecule-1 (ICAM-1) and monocyte chemoattractant protein-1 (MCP-1) were measured by ELISA according to the commercial kits Mouse sICAM-1 Quantikine and Mouse sMCP-1 Quantikine, respectively (R&D Systems, Minneapolis, MN, USA).

### Immunohistochemistry

Immunohistochemical staining of renal issue was performed on formalin-fixed paraffin sections using a microwave-based technique. 4 μm thick sections of the fixed kidneys were dewaxed with xylene, hydrated in graded concentrations of ethanol, and treated with 0.3% hydrogen peroxide for 20 min to quench endogenous peroxidase. The sections were heated in a microwave oven in citrate buffer (0.01 mol/L, PH 6.0) at maximum power (700 W) for 15 min, and then cooled at room temperature for 20 min. The sections were then incubated in 5% blocking serum for 30 min and then in primary antibodies (p-JAK2 and p-STAT3 from Cell Signaling Technology, Beverly, MA, USA; p-STAT1 from Santa Cruz Biotechnology, Santa Cruz, CA, USA) at 4°C overnight. They were subsequently incubated with biotinylated secondary antibodies for 30 min and finally counter-stained with hematoxylin. Sections incubated in the absence of primary antibodies were used as negative controls.

### Western blotting analysis

Frozen renal tissue from each animal was crushed and lysed, subsequently homogenized, then centrifuged at 12,000 rpm for 20 min. The extracted protein was separated in a 10% sodium dodecyl sulfate (SDS)-PAGE, and then electrophoretically transferred to a nitrocellulose membrane (Hybond, Amersham Biosciences, Little Chalfont, UK). Membranes were blocked with 5% non-fat milk powder in TBS for 1 h at room temperature. Blots were further incubated overnight at 4°C with specific antibodies against JAK2, p-JAK2, STAT3, p-STAT3 (1:1000 dilution), STAT1 and p-STAT1 (1:2000 dilution) (Santa Cruz Biotechnology, Santa Cruz, CA, USA), and cleaved caspase 3 (1:1000 dilution; Cell signaling, Beverly, MA). All blots were then washed and incubated with respective horseradish peroxidase coupled secondary antibodies (1:5000 dilution; Jackson Immunoresearch Laboratories, West Grove, PA, USA) at room temperature for 1 h. The protein bands were detected by an enhanced chemiluminescent detective system (Amersham Biosciences UK Ltd., Little Chalfont, UK) and were quantified using the Quantity One software package (Bio-Rad Laboratories, UK). β-actin was presented as internal control to calculate the ratio of optical density, and values were compared with those of sham controls.

### Statistical analysis

All values are expressed as the mean ± the standard error of the mean (SEM). Statistical analysis was carried out using the SPSS 13.0 software (SPSS Inc., USA). Statistical significance was determined by performing a one-way ANOVA followed by Bonferroni’s correction for multiplicity where appropriate. A value of *P* < 0.05 was considered to be statistically significant.

## Results

### Dexmedetomidine treatment improved renal function

**Figure 1:**
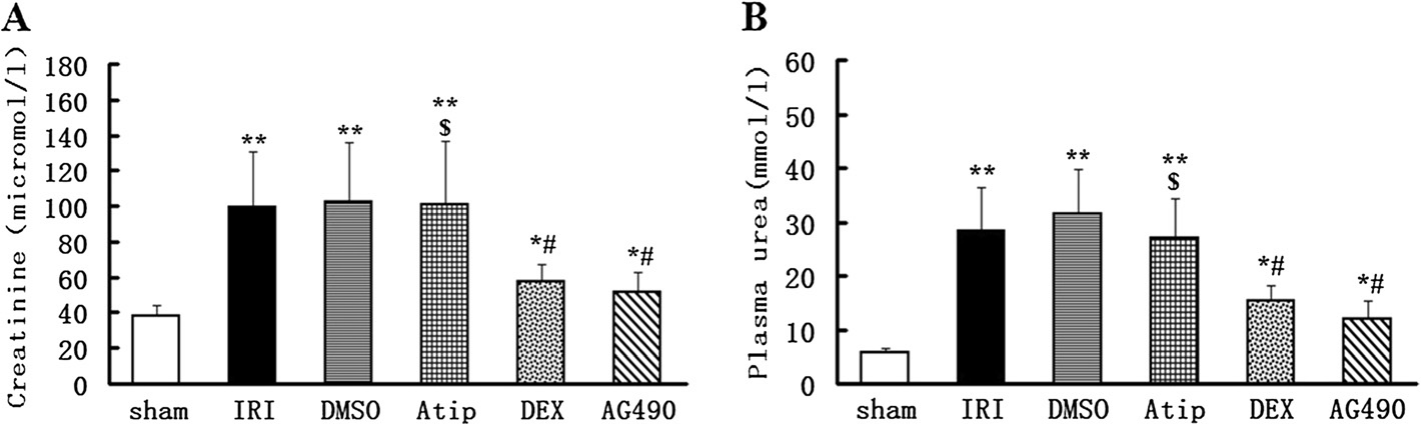
**The Effects of dexmedetomidine on alterations of renal function following renal I/R-induced injury.** Serum creatinine **(A)** and plasma urea **(B)** were measured to assess the renoprotective effect of dexmedetomidine against renal I/R injury in the sham, IRI, DMSO, Atip, DEX and AG490 groups. Data were represented as mean ± SEM (*n* = 8). *P < 0.05 and **P < 0.01 vs. the sham group. ^#^*P* < 0.05 vs. the IRI and DMSO groups. ^$^*P* < 0.05 vs. the DEX group.

All rats survived the experimental period. The rat’s body weight and body temperature during the operation did not differ among groups. In contrast to the sham-operated rats, animals subjected to I/R had dramatic increase in serum creatinine and plasma urea level, indicating renal dysfunction in the IRI and DMSO groups (*P* < 0.01 vs. the sham group). Pre-treatment with dexmedetomidine or AG490 was associated with a smaller increase in serum creatinine and plasma urea level (*P* < 0.05 vs. the sham group and *P* < 0.05 vs. the IRI and DMSO groups) (Figure 1A and B). Atipamezole treatment abolished the protective effects induced by dexmedetomidine (*P* < 0.05 vs. the DEX group).

### Dexmedetomidine treatment attenuated histological lesion

**Figure 2:**
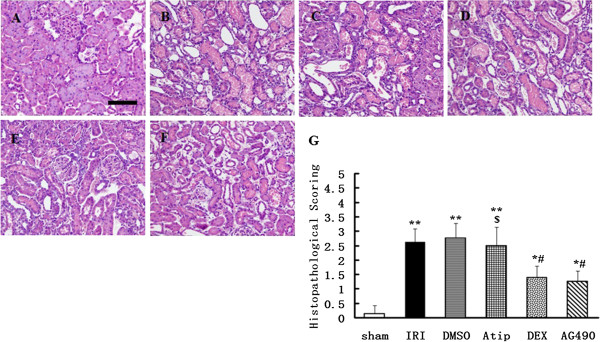
**Effects of dexmedetomidine inhibition on I/R-induced renal injury-histology.** Representative microphotographs were taken from the kidneys of the sham **(A)**, IRI **(B)**, DMSO **(C)**, Atip **(D)**, DEX **(E)** and AG490 **(F)** groups at the time point of 48 h after renal I/R. Histopathological examination was performed using hematoxylin and eosin. Semi-quantitative assessment of the histological lesions based on tubular necrosis **(G)**. Bar = 50 μm. Data were represented as mean ± SEM (*n* = 8). *P < 0.05 and **P < 0.01 vs. the sham group. ^#^*P* < 0.05 vs. the IRI and DMSO groups. ^$^*P* < 0.05 vs. the DEX group.

Representative kidney proximal tubule morphologic changes are presented in Figure 2A-F. As expected, normal morphology of tubular architecture and tubular cells were observed in the sham rats (Figure 2A). In contrast, renal ischemia and reperfusion resulted in severe tubular damage in the IRI group; the destruction included widespread degeneration of tubular architecture, tubular dilation, tubular cell swelling, cellular vacuolization, pyknotic nuclei, severe tubular necrosis and luminal congestion (Figure 2B). In the DMSO and atipamezole groups, tubular damage was comparable to that seen in the IRI group (Figure 2C and D). However, compared with the IRI and DMSO groups, only mild damage in renal histological architecture was seen in the DEX and AG490 groups (Figure 2E and F). The histopathological scores of renal tubular injury are presented in Figure 2G. The scores in the IRI and DMSO groups were significantly higher than that in the sham group (*P* < 0.01) and also in the dexmedetomidine or AG490 groups (*p* < 0.05). However, this injury was significantly attenuated either by dexmedetomidine or AG490 when compared to the IRI and DMSO groups (*P* < 0.05). Renal protective action was abolished when dexmedetomidine treatment was preceded by atipamezole (*P* < 0.05 vs. the DEX group).

### The effect of dexmedetomidine on apoptosis of tubular epithelial cells

**Figure 3:**
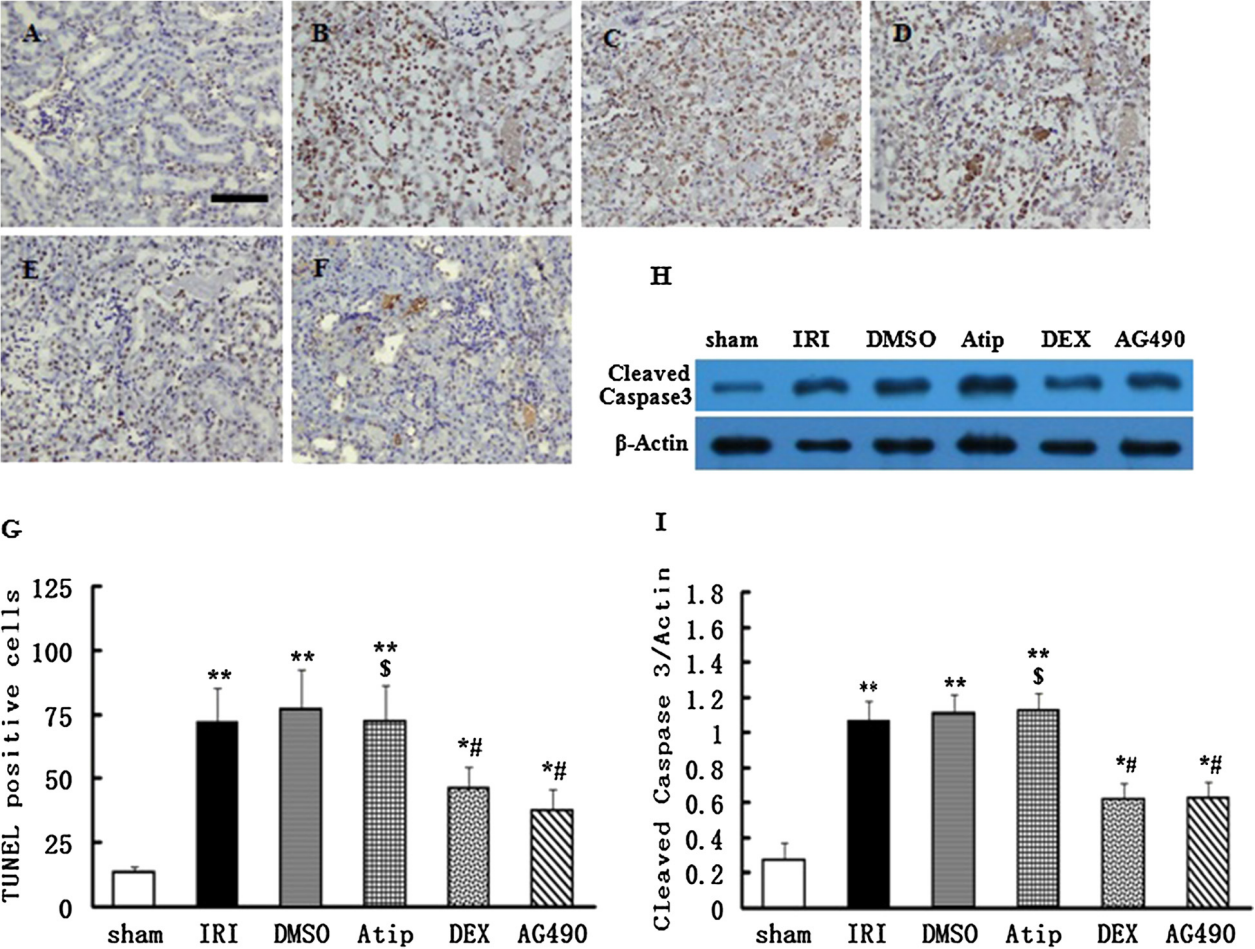
**The effect of dexmedetomidine inhibition on I/R-induced apoptosis of tubular epithelial cells.** Representative microphotographs were taken from the kidneys of the sham **(A)**, IRI **(B)**, DMSO **(C)**, Atip **(D)**, DEX **(E)** and AG490 **(F)** groups at the time point of 48 h after renal I/R in rats. Apoptosis was evaluated by terminal deoxynucleotidyl transferase dUTP nick end (TUNEL) staining. Quantification of TUNEL positive cells was counted following renal I/R **(G)**. Western blots for cleaved caspase 3 expression **(H)** of kidneys were detected after 48 h of renal I/R in all six groups. Densitometry analysis of Western blots for the ratio of cleaved caspase 3/β-Actin **(I)**. Bar = 50 μm. Data were represented as mean ± SEM (*n* = 8). *P < 0.05 and **P < 0.01 vs. the sham group. ^#^*P* < 0.05 vs. the IRI and DMSO groups. ^$^*P* < 0.05 vs. the DEX group.

To evaluate the apoptosis of tubular epithelial cells induced by renal ischemia, a TUNEL assay was used. A large number of apoptotic tubular epithelial cells were visible in the kidneys that were subjected to I/R in the IRI and DMSO groups (*P* < 0.01 vs. the sham group). Either dexmedetomidine or AG490 treatment was associated with the occurrence of apoptosis of tubular epithelial cells (*P* < 0.05 vs. the sham group) which was less than that seen with the IRI and DMSO groups (*P* < 0.05). In the Atip group, atipamezole treatment cancelled the anti-apoptotic effect induced by dexmedetomidine and the number of apoptotic tubular epithelial cells was comparable to those observed in the IRI and DMSO groups (*P* < 0.05 vs. the DEX group) (Figure 3A-G).

### The effects of dexmedetomidine on the expression of caspase 3 in I/R kidneys

In contrast to the sham-operated rats, the I/R procedure significantly increased the expression of caspase 3 in the IRI and DMSO groups (P < 0.01). Pre-treatment with either dexmedetomidine or AG490 was associated with a rise in the expression of caspase 3 (*P* < 0.05 vs. the sham group) which was lower than that seen in the IRI and DMSO groups (*P* < 0.05). In the Atip group, atipamezole pre-treatment suppressed the effect on caspase 3 protein induced by dexmedetomidine (*P* < 0.05 vs. the DEX group) (Figure 3H and I).

### The effects of dexmedetomidine treatment on plasma ICAM-1 and MCP-1 concentrations

**Figure 4:**
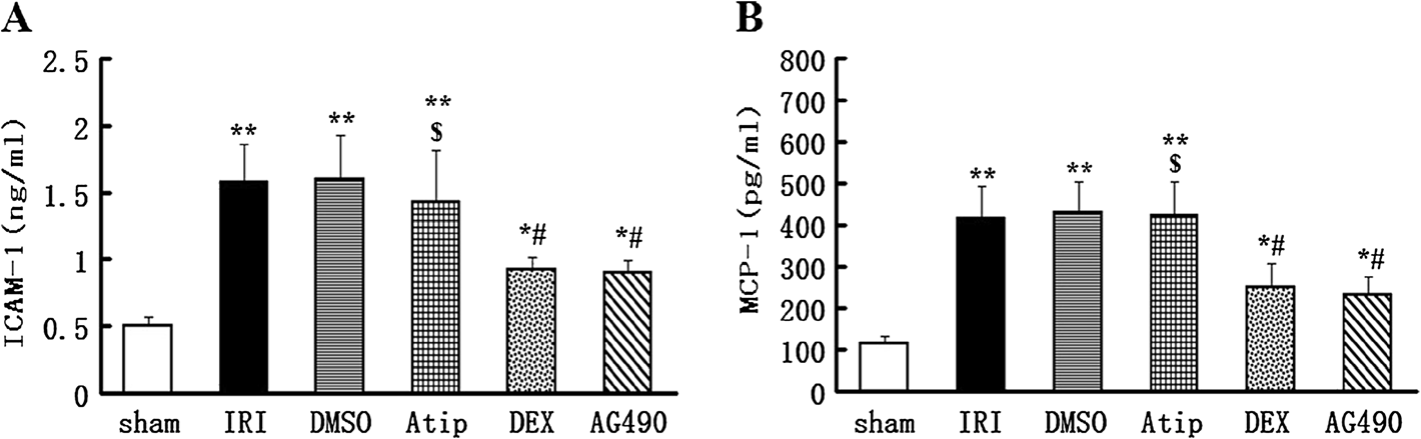
**Effects of dexmedetomidine on the plasma level of adhesion molecule ICAM-1 and chemokine MCP-1 following renal I/R-induced injury.** The concentration of plasma ICAM-1 **(A)** and MCP-1 **(B)** were measured at 48 h following renal I/R injury in the sham, IRI, DMSO, Atip, DEX and AG490 groups. Data were represented as mean ± SEM (*n* = 8). *P < 0.05 and **P < 0.01 vs. the sham group. ^#^*P* < 0.05 vs. the IRI and DMSO groups. ^$^*P* < 0.05 vs. the DEX group.

Rats subjected to I/R had significantly increase in plasma adhesion molecule ICAM-1 and chemokine MCP-1 levels in the IRI and DMSO groups in contrast to the sham-operated rats (*P* < 0.01 vs. the sham group). Pre-treatment with dexmedetomidine or AG490 significantly reduced plasma ICAM-1 and MCP-1 levels (*P* < 0.05 vs. the sham group and *P* < 0.05 vs. the IRI and DMSO groups). Atipamezole abolished the effects on the level of plasma ICAM-1 and MCP-1 induced by dexmedetomidine in the Atip group (*P* < 0.05 vs. the DEX group) (Figure 4A and B).

### Dexmedetomidine inhibited renal p-JAK2, p-STAT1 and STAT3 protein expressions

**Figure 5:**
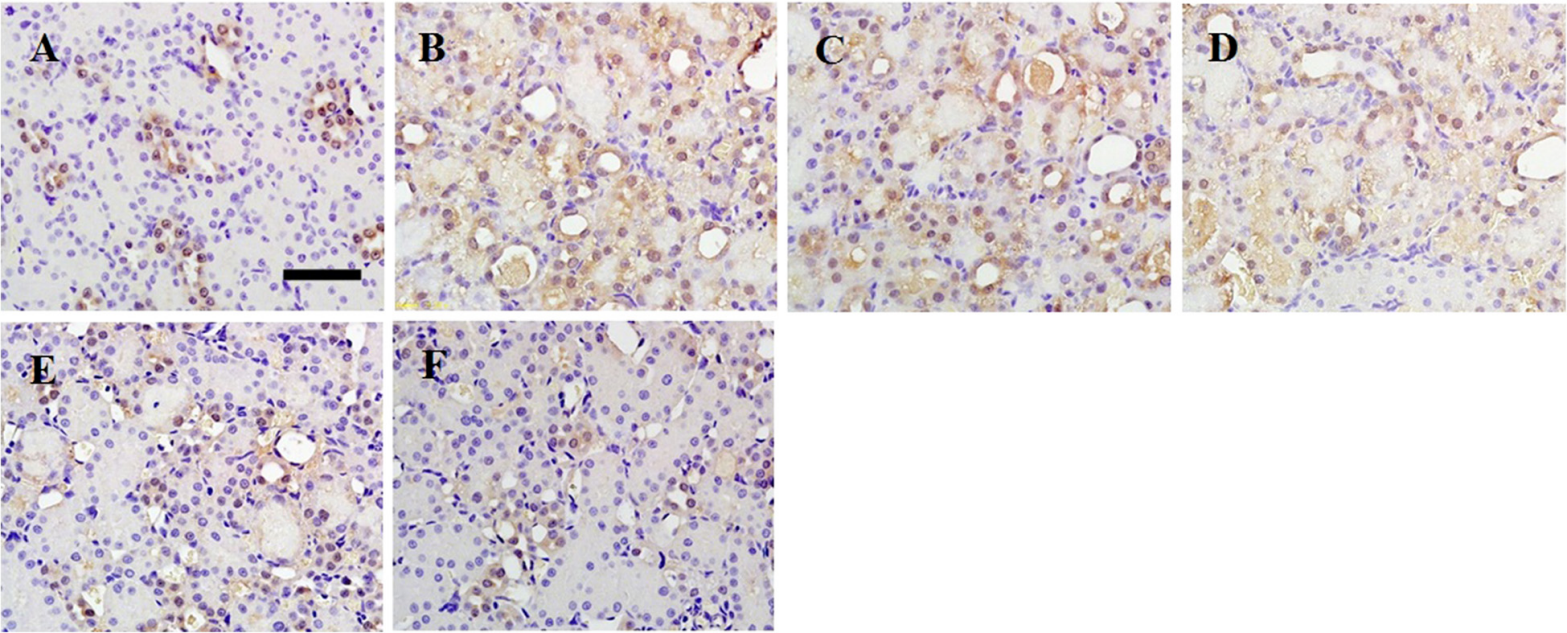
**The effect of dexmedetomidine on the expression of p-JAK2 in the kidneys following renal I/R-induced injury.** Immunohistochemical staining of p-JAK2 was performed on formalin-fixed paraffin-embedded kidneys of the sham **(A)**, IRI **(B)**, DMSO **(C)**, Atip **(D)**, DEX **(E)** and AG490 **(F)** groups at 48 h following renal I/R. Bar = 50 μm. *n* = 8 per group.

**Figure 6:**
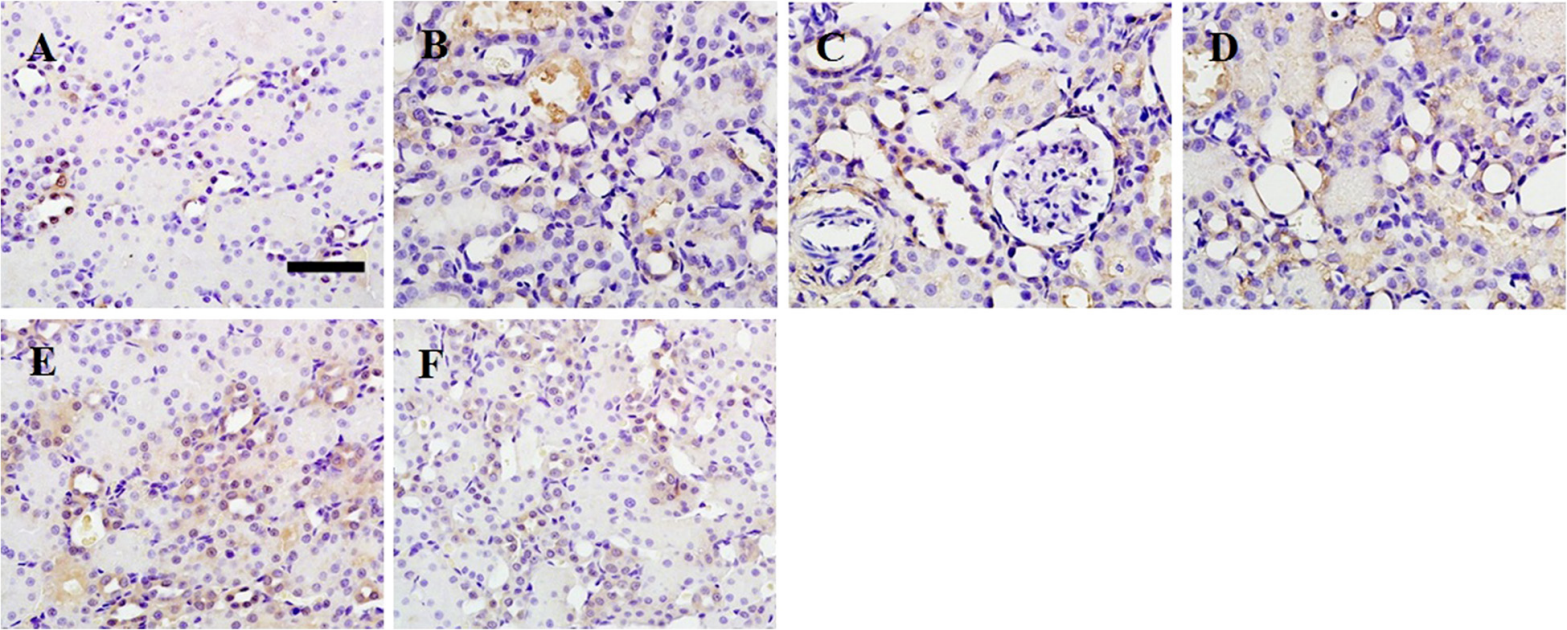
**The effect of dexmedetomidine on the expression of p-STAT1 in the kidneys following renal I/R-induced injury.** Immunohistochemical staining of p-STAT1 was performed on formalin-fixed paraffin-embedded kidneys of the sham **(A)**, IRI **(B)**, DMSO **(C)**, Atip **(D)**, DEX **(E)** and AG490 **(F)** groups at 48 h following renal I/R. Bar = 50 μm. *n* = 8 per group.

**Figure 7:**
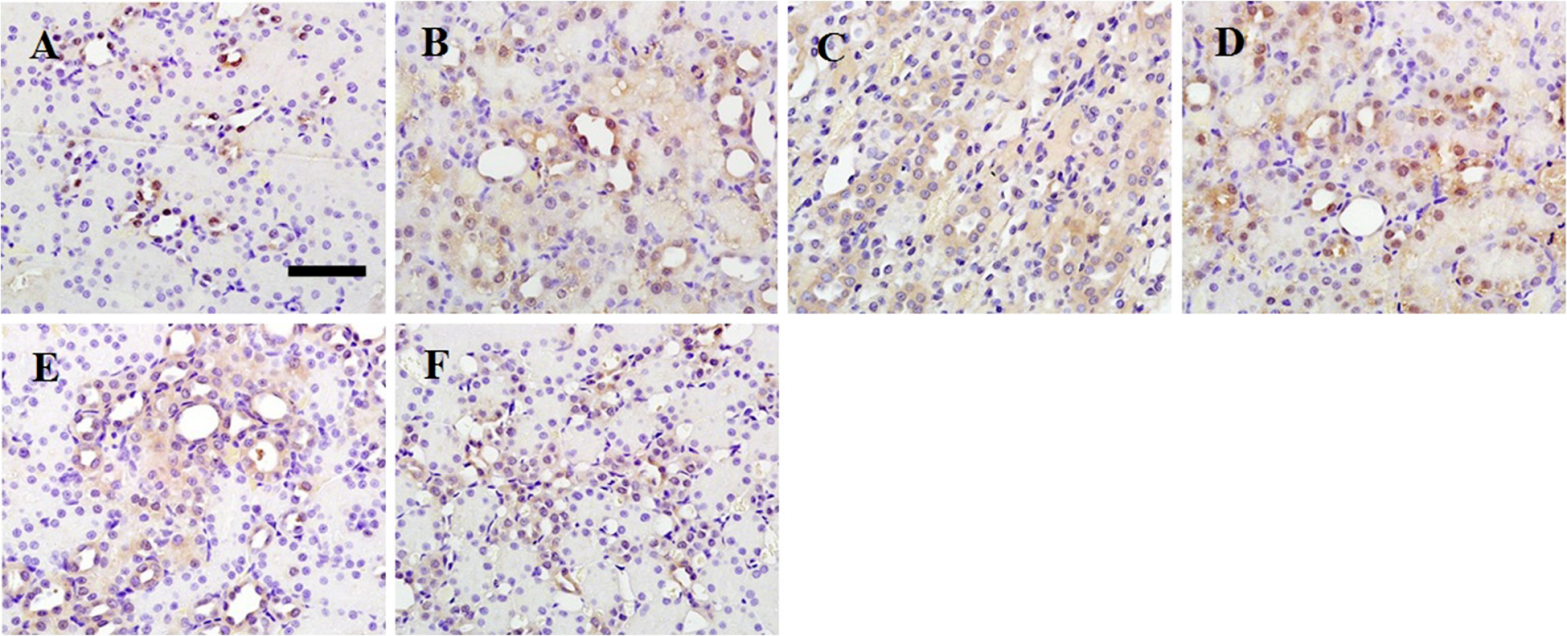
**The effect of dexmedetomidine on the expression of p-STAT3 in the kidneys following renal I/R-induced injury.** Immunohistochemical staining of p-STAT3 was performed on formalin-fixed paraffin-embedded kidneys of sham **(A)**, IRI **(B)**, DMSO **(C)**, Atip **(D)**, DEX **(E)** and AG490 **(F)** groups at 48 h following renal I/R. Bar = 50 μm. *n* = 8 per group.

P-JAK2, p-STAT1 and p-STAT3 proteins were mainly expressed in renal tubular epithelial cells and stromal vascular endothelial cells. Normal rat kidneys had weak expressions of P-JAK2, p-STAT1 and p-STAT3 proteins (Figure 5, 6 and 7A, respectively). Immunohistochemical staining showed augmented expressions of P-JAK2, p-STAT1 and p-STAT3 proteins in the kidneys of the IRI and DMSO groups (Figure 5, 6 and 7B and C, respectively). The expressions of these three proteins significantly decreased in the kidneys of the DEX and AG490 groups (Figure 5, 6 and 7E and F, respectively). Atipamezole treatment abolished the effects on the inhibition of P-JAK2, p-STAT1 and p-STAT3 proteins induced by dexmedetomidine (Figure 5, 6 and 7D, respectively).

### Blockage of JAK/STAT signaling by dexmedetomidine

**Figure 8:**
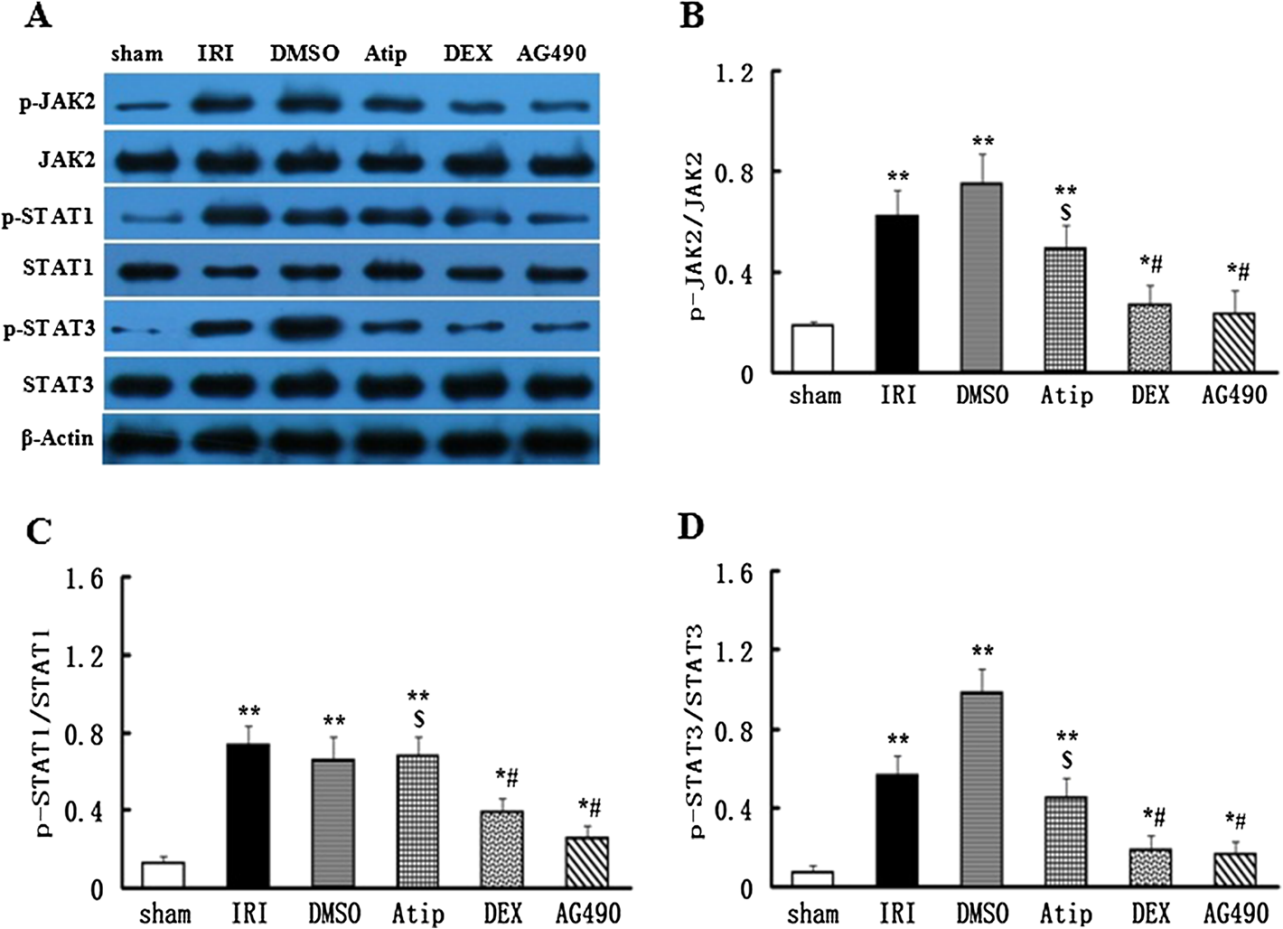
**Dexmedetomidine inhibited the phosphorylations of JAK2, STAT1 and STAT3.** Representative Western blots for the phosphorylations of JAK2, STAT1, STAT3 and total JAK2, STAT1, STAT3 expression **(A)** of the kidneys were detected after 48 h of renal I/R in the sham, IRI, DMSO, Atip, DEX and AG490 groups. Densitometry analysis of Western blots for the ratio of p-JAK2/JAK2 **(B)**, p-STAT1/STAT1 **(C)** and p-STAT3/STAT3 **(D)**. Data were represented as mean ± SEM (*n* = 8). *P < 0.05 and **P < 0.01 vs. the sham group. ^#^*P* < 0.05 vs. the IRI and DMSO groups. ^$^*P* < 0.05 vs. the DEX group.

To verify that dexmedetomidine exerted its renoprotective effects via inhibiting JAK/STAT signaling, we performed western blot to analyse the phosphorylations of JAK2, STAT1 and STAT3. In the kidney of sham-operated rats, there is a low grade of phosphorylation for JAK2. The expression of p-JAK2 protein significantly increased in contrast to total JAK2 in the kidney subjected to renal I/R in the IRI and DMSO groups (*P* < 0.01 vs. the sham group), but the expression of total JAK2 retain the level of the sham-operated rats. Treatment with dexmedetomidine or AG490 *in vivo* resulted in reducing the phosphorylation of JAK2 (*P* < 0.05 vs. the sham group and *P* < 0.05 vs. the IRI and DMSO groups). The dexmedetomidine-induced inhibition of the expression of p-JAK2 was abolished by atipamezole in the Atip group (*P* < 0.05 vs. the DEX group) (Figure 8A and B). In the mean time, p-STAT1 and p-STAT3, downstream molecules of JAK2 cascade, were also significantly increased in the IRI and DMSO groups (*P* <0.01 vs. the sham group). The phosphorylation of STAT1 and STAT3 was inhibited by either dexmedetomidine or AG490 treatment (*P* < 0.05 vs. the sham group and *P* <0.05 vs. the IRI and DMSO groups) The expressions of p-STAT1 and p-STAT3 in the Atip group were comparable to those seen in the IRI and DMSO groups and higher than those in the DEX group (*P* < 0.05 vs. the DEX group) (Figure 8A, C and D).

## Discussion

Dexmedetomidine has been described as a useful, safe adjunct in many clinical applications. It has been found that dexmedetomidine may increase urine output by considerably redistributing of cardiac output, inhibiting vasopressin secretion and maintaining renal blood flow and glomerular filtration [16-18]. Hsing *et al*. suggested that dexmedetomidine reduced sepsis-induced AKI by *in vitro* and *in vivo* experimentation [29]. Dexmedetomidine is also benefit for the kidney suffering from renal ischemia and reperfusion injury which may develop AKI [14]. Therefore, dexmedetomidine pre-treatment may be of benefit to patients with low preoperative eGFR (<60 ml/min or <40 ml/min) undergoing vascular surgery (AAA, EVAR); cardiology interventions (TAVI, complex angioplasty) or cardiac surgery [30,31]. These patients are known to have a high risk of developing postoperative renal failure, but we are unaware of any clinical studies to assess this. In the present study, the renoprotective effect of dexmedetomidine, a highly selective α_2_-adrenoreceptor agonist, was shown by an improved post-ischemic renal functional recovery, attenuated histological lesions, reduced number of apoptotic tubular epithelial cells and down-regulation of the adhesion molecule ICAM-1 and chemokine MCP-1. The major new findings of this study, in which we systematically examined the spatial activation of JAK/STAT signaling pathway in the kidney following renal ischemia, was that dexmedetomidine treatment inhibited the phosphorylation of JAK2, accompanied by down-regulation in the phosphorylation of downstream protein STAT1 and STAT3. These results indicate that the renoprotective effect of dexmedetomidine is at least partially dependent on inhibiting the activation of the JAK/STAT signaling pathway.

In line with previous studies, our data also showed that dexmedetomidine’s renoprotective properties have largely been attributed to its agonist actions at α_2_-adrenoreceptors. Its protective effects against renal I/R injury, which are abolished by α_2_-adrenoreceptor antagonists, have been reported in different animal models. When administrated before ischemia, dexmedetomidine improves renal function recovery, reduces the number of apoptotic tubular epithelial cells and attenuates renal tissue necrosis and histological lesions in a rat acute I/R injury model [14,17,32]. It has been recently found that dexmedetomidine reduces systemic levels of interleukin- 6 (IL-6), tumor necrosis factor α (TNF-α) and high mobility group box 1 (HMGB1) following lipopolysaccharide infusion or sepsis in animals, indicating its anti-inflammatory effects against renal I/R injury [14]. We did not explore the well described anti-inflammatory properties in this study. However, we further demonstrated that dexmedetomidine pre-treatment mediates significant attenuation in the expression of the adhesion molecule ICAM-1 and the chemokine MCP-1 in an *in vivo* renal I/R model. We, for the first time, investigated the relationship between dexmedetomidine’s renoprotective action and the activation of JAK/STAT signaling pathway, which is associated with signaling cascades induced by renal I/R injury. The phosphorylation of JAK2, STAT1 and STAT3, reflecting activation, were significantly potentiated after an ischemia and reperfusion procedure [33]. Previous studies showed conflicting results about the critical role of JAK/STAT signaling pathway and the therapeutic effect of its inhibitor (AG490) in regulating I/R injury. Sharples *et al*. suggested that the JAK2-specific inhibitor AG490 blocked the reduction in cell death observed with erythropoietin (EPO) in a dose-dependent manner in an *in vitro* study [34]. AG490 or its analogs could abolish the renoprotective effect of ischemic or pharmacological preconditioning [35] and promote apoptosis through down-regulating phosphorylation of STAT1 and STAT3 [36]. In contrast, Ruetten H and Thiemermann C found that AG490 prevented the multiple organ dysfunction induced by endotoxic shock [37]. Pre-treatment or immediate post-ischemia treatment of AG490 significantly ameliorated renal injury via the inactivation of JAK/STAT signaling pathway in a recent study [19]. We found that AG490 down-regulated its downstream molecules, STAT1 and STAT3, but this was associated with improved renal function and attenuated histological lesions against renal I/R injury. In addition, dexmedetomidine significantly reduced the expression of phosphorylated forms of JAK2, STAT1 and STAT3, and provided the same renoprotective effect as AG490 in our study. Our results indicated that dexmedetomidine’s renoprotective effect was at least partially dependent on inhibiting the activation of JAK/STAT signaling pathway induced by renal I/R, which may contribute to ameliorating renal injury. The present study suggested that dexmedetomidie and tyrphostin AG490 acted on the same cascade.

To further elucidate whether down-regulation of JAK/STAT signaling pathway is involved in the renoprotective properties induced by dexmedetomidine in an *in vivo* I/R injury model, we performed additional experiments after taking into consideration the following aspects. First, consistent with previous studies [38-40], renal I/R injury was accompanied with a dramatic increase in plasma level of the adhesion molecule ICAM-1. Second, AG490 significantly decreased systemic level of ICAM-1, while also inhibiting the phosphorylation of JAK2, STAT1 and STAT3 in a renal I/R injury rat. Thirdly, pre-treatment with dexmedetomidine conferred the same effect as AG490 on ICAM-1 according to our findings. The adhesion molecule ICAM-1 is responsible for renal I/R-induced recruitment of granulocyte and macrophage infiltration. Recent evidences suggest that treatment with anti-ICAM-1 monoclonal antibody, ICAM-1 antisense oligodeoxyribonucleotides and ablation of the ICAM-1 gene result in less pathological and functional damage in the rat subjected to renal I/R [19,38-40]. ICAM-1 expression is transcriptionally regulated by several pro-inflammatory cytokines including IFN-γ via the JAK/STAT signaling pathway in a STAT-dependent fashion [41]. It is likely that the down-regulation of ICAM-1 expression mediated by the inactivation of JAK/STAT pathway is liable for dexmedetomidine’ renoprotective property against renal I/R injury according to our results. Our findings further suggest that either dexmedetomidine or AG490 pre-treatment is responsible for the inhibition of granulocyte and macrophage infiltration, subsequently ameliorating renal injury following I/R *in vivo*.

A growing body of evidence indicates that the inflammatory response, associated with pro-inflammatory cytokines IL-1β, TNF-α and chemotactic cytokine MCP-1, plays a major role in renal dysfunction following ischemia and reperfusion [42]. It has been found that α_2_-adrenoreceptor agonist might attenuate the increase in plasma level of IL-1β, TNF-α and improve survival successfully after caecal ligation and puncture (CLP)-induced sepsis [43], and reduce the incidence of sepsis-induced AKI by decreasing TNF-α and MCP-1 [29]. MCP-1 is an inflammatory molecule whose synthesis is regulated by several signaling pathways. It has been demonstrated that MCP-1 gene induction is blocked by protein kinase A (PKA), p38 mitogen activated protein kinase (MAPK) and JAK-STAT inhibitors [42,44,45]. Toll-like receptor 2 (TLR2)-mediated MCP-1 expression decreased via blockade of the JAK/STAT signaling pathway [46]. The up-regulation of MCP-1, which is responsible for the inflammatory cascade response, is mediated by the activation of IL-6-induced JAK/STAT pathway [45]. However, the role of MCP-1 in dexmedetomidine’s renoprotection and its molecule mechanism are not unknown. In the present study, dexmedetomidine significantly attenuated the I/R-induced up-regulation of MCP-1, consistent with its inhibitory effects on JAK2, STAT1 and STAT3 activation. Its inhibitory effects on MCP-1 and JAK/STAT pathway were similar to the selective JAK2 inhibitor AG490. Our results indicate that down-regulation of MCP-1 expression is associated with *in vivo* inactivation of JAK/STAT signaling pathway following dexmedetomidine pretreatment in a renal I/R model.

Apoptosis plays as a major role of cell death in the destruction of renal proximal tubule following renal I/R [47]. To confirm the hypothesis that JAK/STAT signaling pathway inhibition by AG490 is involved in regulating apoptotic process in the tubular epithelial cells following I/R insult, the TUNEL staining method was performed and cleaved caspase 3 protein expression was detected. The dexmedetomidine-induced inactivation of JAK/STAT was observed with a reduced number of apoptotic tubular epithelial cells and a decrease in pro-apoptotic factor cleaved caspase 3, the same effects as AG490 in the present study. According to previous studies, JAK/STAT signaling pathway mediates cell apoptotic signals through the induction of anti-apoptotic bcl-2 and the inhibition of caspase 3 protein expression [19,36,48]. Indeed, some studies have documented that dexmedetomidine significantly attenuates apoptosis in the brain, intestine, heart, testis, neutrophils and kidney during *in vivo* or *in vitro* experiments [9-14,49,50]. Our results showed that AG490 significantly suppressed apoptosis and reduced the expression of cleaved caspase 3 protein following renal I/R, which strongly indicate a possible interaction of the JAK/STAT and the anti-apoptotic pathways. Additionally, dexmedetomidine-induced anti-apoptosis is regulated by the JAK/STAT pathway, contributing to its renoprotective effects on renal injury.

In summary, renal I/R injury leads to the deterioration of renal function and histological lesions, enhanced apoptosis of tubular epithelial cells and the expression of protein caspase 3, accompanied by up-regulation of the adhesion molecule ICAM-1 and chemokine MCP-1. We demonstrate that dexmedetomidine treatment results in a partial, but significant, attenuation of renal damage induced by I/R injury through the inactivation of JAK/STAT signaling pathway in an *in vivo* model. Atipamezole abolished the renoprotective effect that was conferred by dexmedetomidine administrated before ischemia. Furthermore, inhibiting the JAK/STAT pathway with selective JAK2 inhibitor AG490 ameliorates the pathogenesis of renal I/R injury. Similar to the effects of AG490, dexmedetomidine produces its renoprotective effect by regulating the activation of the JAK/STAT signaling pathway, indicating intervention targeted at this signal transduction pathway may have therapeutic potential for treatment of perioperative AKI.

## Conclusions

Our studies showed that dexmedetomidine protects kidney against I/R injury, at least in part, through its inhibitory effects on injury-induced activation of JAK/STAT signaling pathway. If our data can be extrapolated to clinical setting, then dexmedetomidine may therefore serve as a clinical strategy to treat/prevent perioperative renal I/R injury.

## Abbreviations

AKI: Acute kidney injury; I/R: Ischemia and reperfusion; IRI: I/R injury; DEX: Dexmedetomidine; Atip: Atipamezole; JAKs: Tyrosine kinases; STATs: transcription factors; ERK: Extracellular regulated protein kinases; TUNEL: Terminal deoxynucleotidyl transferase (TDT)-mediated digoxigenin-labelled UTP nick end labelling; DAB: Diaminobenzidine; ICAM-1: Intercellular adhesion molecule-1; MCP-1: Monocyte chemoattractant protein-1; IL-6: Interleukin- 6; TNF-α: Tumor necrosis factor α; HMGB1: High mobility group box 1; PKA: Protein kinase A; TLR2: Toll-like receptor 2.

## Competing interest

The authors declared that they have no competing interest.

## Authors’ contributions

HGB, DQM and YNS conceived the study design. YNS, LX and CHL performed the experiment. LH, HWS and YZ participated in the data analysis and interpretation. DQM and AV helped to draft and revise the manuscript. JSW and XBY performed pathological analysis and immunohistochemical study. All authors read and approved the final manuscript.
